# Supplementary material for: Tetraspanin CD9 is Regulated by miR-518f-5p and Functions in Breast Cell Migration and In Vivo Tumor Growth
Source: Cancers (Basel). 2020 Mar 26;12(4):795. doi: 10.3390/cancers12040795 (PMC7226392; doi:10.3390/cancers12040795)
Supplement: Supplementary file 1 [file cancers-12-00795-s001.pdf]

Supplementary Materials: Tetraspanin CD9 is Regulated by miR-518f-5p and Functions in Breast Cell Migration and In Vivo Tumor Growth

Danielle R. Bond, Richard Kahl, Joshua S. Brzozowski, Helen Jankowski, Crystal Naudin, Mamta Pariyar, Kelly A. Avery-Kiejda, Christopher J. Scarlett, Claude Boucheix, William J. Muller, Leonie K. Ashman, Murray J. Cairns, Séverine Roselli and Judith Weidenhofer

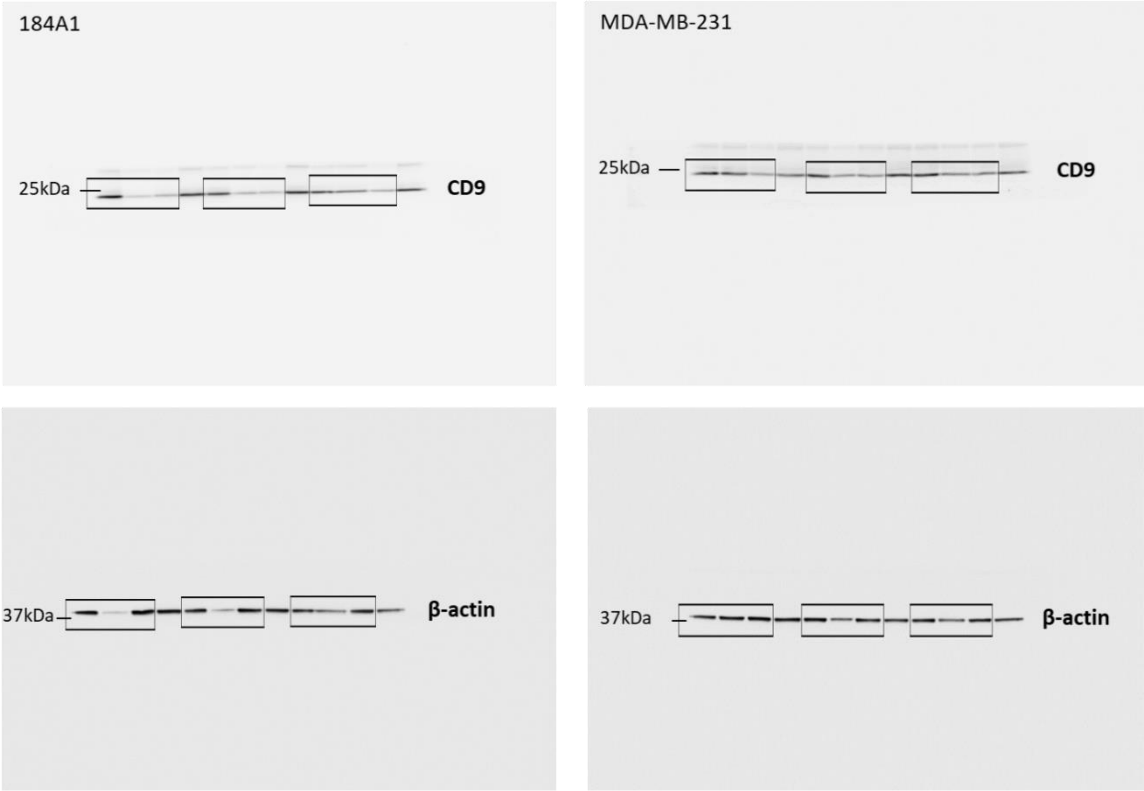

184A1 (Beta-actin)

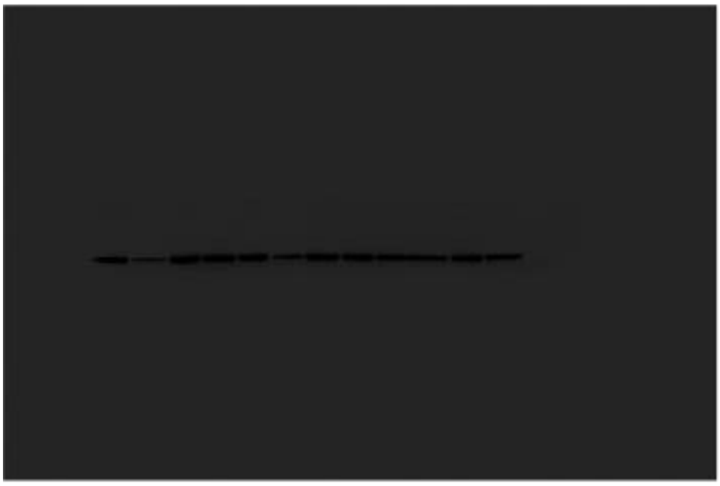

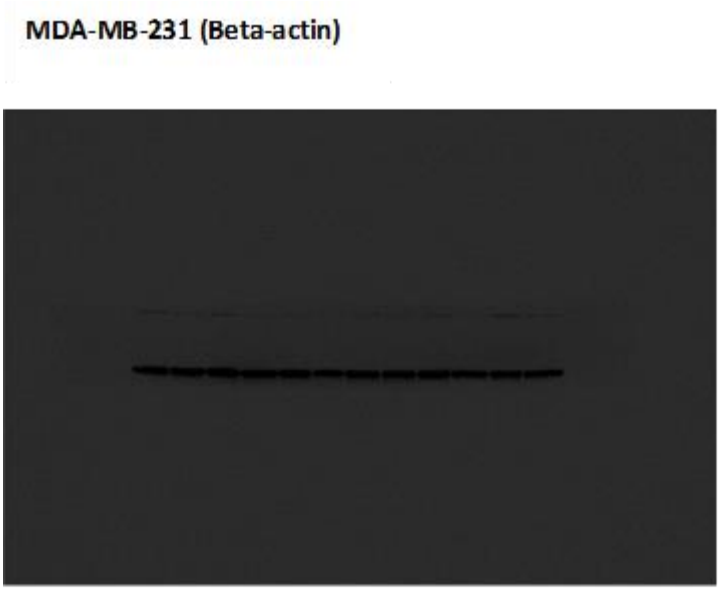

Figure S1. Whole western blotting images from Figure 1. Bands from 184A1 protein lysates (left) and MDA-MB-231 protein lysates (right), CD9 bands (top) and beta-actin (bottom). The boxes contain the bands used for quantitation for lysates from mock, NTC and miR-518f-5p mimic, respectively. Lanes 4, 8 and 12 contain lysates from miR-4289 mimic transfected cell lines that served as a positive control.

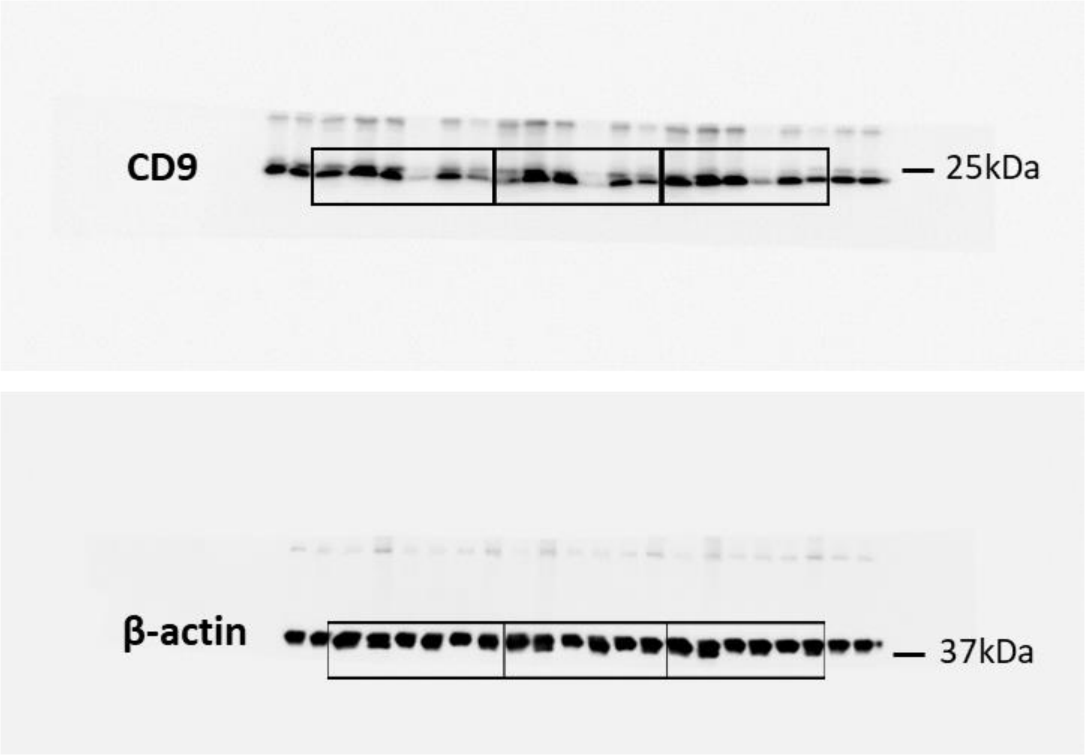

Figure S2. Whole western blotting images from figure 3. Boxes contain bands for the different breast cell lysates in the following order: HMEC, 184A1, MCF7, T-47D, SKBR3 and MDA-MB-231. Lanes 1, 2, 21 & 22 contain the positive control RWPE-1 protein lysate.

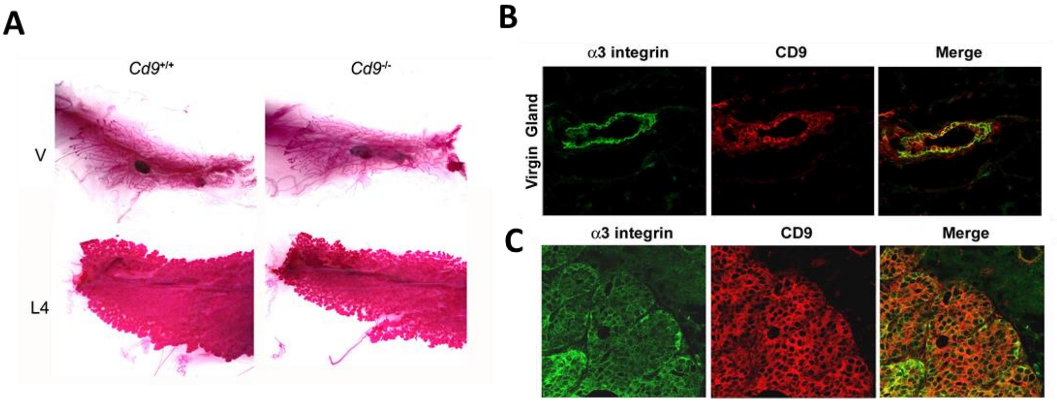

Figure S3. *Cd9* ablation does not affect mammary gland development and CD9 is expressed in the normal mouse mammary gland and in PyMT tumours. A. Whole mount FVB/N *Cd9*$^{+/+}$ and *Cd9*$^{-/-}$ mouse mammary glands ($n = 2$ in each group) stained with Carmine Alum. V: Virgin; L4: Lactation Day 4. B. Co-labelling of 6-week old virgin gland with CD9 and α3 integrin antibodies. C. Co-labelling of 10-week old PyMT tumours with CD9 and α3 integrin antibodies.

© 2020 by the authors. Licensee MDPI, Basel, Switzerland. This article is an open access article distributed under the terms and conditions of the Creative Commons Attribution (CC BY) license (http://creativecommons.org/licenses/by/4.0/).
